# Novel Severe Hemophilia A Mouse Model with Factor VIII Intron 22 Inversion

**DOI:** 10.3390/biology10080704

**Published:** 2021-07-23

**Authors:** Jeong Pil Han, Dong Woo Song, Jeong Hyeon Lee, Geon Seong Lee, Su Cheong Yeom

**Affiliations:** 1 Graduate School of International Agricultural Technology and Institute of Green BioScience and Technology, Seoul National University, 1447 Pyeongchang-ro, Daehwa, Pyeongchang 25354, Korea; pil1426@snu.ac.kr (J.P.H.); ljhljh7506@snu.ac.kr (J.H.L.); lgs6245@snu.ac.kr (G.S.L.); 2 Toolgen Inc., Geumcheon-gu, Seoul 08501, Korea; dw.song@toolgen.com; 3 WCU Biomodulation Major, Department of Agricultural Biotechnology, Seoul National University, Gwanank-gu, Seoul 08826, Korea

**Keywords:** factor VIII, hemophilia A, CRISPR/Cas9, structural variation

## Abstract

**Simple Summary:**

Recently, innovative gene therapy has been developing toward functional restoration by gain of function or gene correction. Hemophilia is a representative genetic disorder with many human patients and is considered a candidate disease for gene therapy. The most frequent severe hemophilia A is caused by inversion mediated structural variation of the human *F8* gene. Nevertheless, a mouse model with *F8* intron 22 inversion is not developed yet. This study presents a novel hemophilia A mouse model with 319 kb inversion and severe coagulation disorder and could be utilized in future gene correction preclinical trials.

**Abstract:**

Hemophilia A (HA) is an X-linked recessive blood coagulation disorder, and approximately 50% of severe HA patients are caused by *F8* intron 22 inversion (F8I22I). However, the F8I22I mouse model has not been developed despite being a necessary model to challenge pre-clinical study. A mouse model similar to human F8I22I was developed through consequent inversion by CRISPR/Cas9-based dual double-stranded breakage (DSB) formation, and clinical symptoms of severe hemophilia were confirmed. The F8I22I mouse showed inversion of a 391 kb segment and truncation of mRNA transcription at the *F8* gene. Furthermore, the F8I22I mouse showed a deficiency of FVIII activity (10.9 vs. 0 ng/mL in WT and F8I22I, *p* < 0.0001) and severe coagulation disorder phenotype in the activated partial thromboplastin time (38 vs. 480 s, *p* < 0.0001), in vivo bleeding test (blood loss/body weight; 0.4 vs. 2.1%, *p* < 0.0001), and calibrated automated thrombogram assays (Thrombin generation peak, 183 vs. 21.5 nM, *p* = 0.0012). Moreover, histological changes related to spontaneous bleeding were observed in the liver, spleen, and lungs. We present a novel HA mouse model mimicking human F8I22I. With a structural similarity with human F8I22I, the F8I22I mouse model will be applicable to the evaluation of general hemophilia drugs and the development of gene-editing-based therapy research.

## 1. Introduction

Mutations in genes such as missense and nonsense mutations, splicing, in-frame insertions or deletions, frameshift insertions or deletions, and structural variations can result in genetic disorders. Notably, genetic structural variations such as those observed in *Factor VIII* (*F8*, hemophilia A), *Iduronate 2-sulfatase* (Hunter syndrome), *MutS Homolog 2* (Lynch syndrome), and *Echinoderm microtubule-associated protein like 4*-*anaplastic lymphoma kinase rearrangement* (non-small cell lung cancer) are directly linked as causal factors to incurable diseases [1]. Hemophilia A (HA) is an X-linked recessive blood coagulation disorder caused by mutations in the coagulation *F8* gene, which encodes for the blood coagulation factor, FVIII. The estimated incidence of HA is 1:5000 males in the United States [2,3]. Inversions, large deletions, nonsense mutations, and missense mutations in the *F8* gene can cause HA. HA is classified into mild, moderate, and severe types, depending on FVIII activity [4]. Approximately 50% of HA patients are classified as the severe type, and approximately 50% of these severe HA cases are caused by an inversion in the intron 22 of the *F8* gene (F8I22I) [5].

At present, the most common treatment for severe HA is prophylaxis, during which the coagulation factor is maintained at a concentration of 1% or more by intravenous FVIII protein infusion [6]. Despite the guaranteed efficacy of protein-based therapy in HA, it presents several limitations, including the short half-life of the protein and the potential generation of anti-FVIII antibodies called inhibitors [7,8]. Adeno-associated virus (AAV) gene therapy has been clinically tested and has demonstrated efficacy in restoring the deficient clotting factors. However, this approach has a limitation of transient expression and is not suitable for patients with inhibitors, which comprise approximately 30% of the HA cases [9]. To overcome the various limitations of existing treatment methods and provide a permanent treatment for HA, gene correction technology must be considered as a new option in the future.

Considerable efforts have been made to improve HA treatment, and many preclinical trials have been carried out on various animal models. The most commonly used animal models for HA are mice, dogs, and pigs. Almost all animal models are *F8* knockout (KO) models generated by genome editing [10]. Although two dog strains with F8I22I were reported [11,12], the F8I22I mouse model has not been developed despite being a necessary model to be used in pre-clinical studies. F8I22I occurs spontaneously by non-allelic meiotic recombination between the *F8* intron 22 and either of two inversely oriented homology regions, located 500 kb and 575 kb away in humans [13].

Currently, clustered regularly interspaced short palindromic repeats (CRISPR)/CRISPR associated protein 9 (Cas9) is widely used to generate mutant mice [14]. In a previous report, precise inversion of DNA fragments ranging in size from a few tens of bp to hundreds of kb could be generated in mice using the CRISPR/Cas9 with two single guide RNAs (sgRNAs) [15]. In this study, we electroporated two sgRNAs and the Cas9 protein into mouse single-cell embryos to invert a 319 kb fragment of the *F8* gene. Using this method, we produced an F8I22I mouse model with an inversion similar to that of human HA patients. Furthermore, we analyzed the hemophilic phenotypes of the F8I22I mouse model.

## 2. Materials and Methods

### 2.1. sgRNA Preparation

Streptococcus pyogenes Cas9 (SpCas9) was used to induce DNA breakage. Ten single guide RNAs (sgRNAs) were designed using an RNA-guided engineered nuclease (RGEN) online tool (www.rgenome.net (accessed on 15 December 2014)) on the *F8* intron 22 and the intragenic region 319 kb away in the direction of the telomere. To assess the cleavage potential of the designed sgRNAs, each sgRNA and Cas9 protein were transfected into NIH3T3 cells using a Neon electroporator (Thermo Fisher Scientific, Waltham, MA, USA). The cells were harvested after 48 h of incubation, DNA was extracted, and deep sequencing was conducted on the resulting PCR amplicons using MiSeq (Illumina, San Diego, CA, USA). The indel ratio of the amplified target region was analyzed using an online Cas-Analyzer (www.rgenome.net (accessed on 15 December 2014)) [16]. An indel appearing 3 bp upstream of the 5′-NGG-3′ protospacer adjacent motif (PAM) was considered as a mutation caused by ribonucleoprotein and the indel efficiency was calculated by comparing the read sequence and reference sequence. Thus, high-efficiency sgRNAs were selected for subsequent recombinant animal development.

### 2.2. Mutant Mouse Generation

C57BL/6 mice were purchased from Koatech (Pyeongtaek, Korea). For estrus synchronization and superovulation, pregnant females were injected with 5 IU of serum gonadotropin (Prospec Bio, East Brunswick, NJ, USA) and 5 IU of human chorionic gonadotropin (hCG) (Prospec) at 48 h intervals. The females were then mated with sperm donor mice. After collecting embryos from the oviduct, embryos were washed three times with Opti MEM I medium (Invitrogen, Carlsbad, CA, USA). Approximately 50 embryos were transferred and into the electrode with an electroporation buffer. The final concentration of the electroporation solution consisted of 200 ng/μL of SpCas9 protein (Toolgen Inc., Seoul, Korea) and 50 ng/μL of each sgRNA. The electroporation pulse conditions were as follows: 7 cycles at 30 V and 3 ms ON and 97 ms OFF. After washing using M2 medium (MTI-GlobalStem, Rockville, MD, USA), the embryos were transferred into the oviduct of the recipient female. Genotyping was conducted using DNA from toe clips, and PCR and Sanger sequencing-based genotyping was conducted. 12–16 week-old mature adult male mice were used for subsequent experiments. This study was approved by the Institutional Animal Care and Use Committees of Seoul National University (SNU-160721-2 and 170522-1) and was conducted in accordance with the approved guidelines.

### 2.3. ELISA and Chromogenic Assay

FVIII protein concentrations in the blood were measured by enzyme-linked immunosorbent assay(ELISA) using a Mouse Factor VIII ELISA kit (MyBioSource, San Diego, CA, USA). Whole blood was collected in 3.2% sodium citrate, and plasma was freshly separated by centrifugation at 1500× *g* for 10 min at room temperature. ELISA was performed according to the manufacturer’s instructions. Next, FVIII activity was measured using a Factor VIIIa Activity Assay Kit (Abcam, Cambridge, MA, USA). Fluorescence intensity (excitation/emission = 360/450 nm) was measured using Cytation 5 (BioTek, Winooski, VT, USA). The FVIII activity was calculated by applying the measured fluorescence intensity values to the standard curve values.

### 2.4. Prothrombin Time and Activated Partial Thromboplastin Time Test

Blood (450 µL) was collected and added 50 µl of a 3.2% sodium citrate solution (Medicago, Durham, NC, USA) from the inferior vena cava, and plasma was collected after centrifugation at 1500 × *g* for 10 min at 4°C. In the microplate-based activated partial thromboplastin time (aPTT) analysis, 30 µL of plasma and aPTT reagent (ThermoFisher Scientific, Waltham, MA, USA) were mixed in 96-wells microplates and incubated at 37 °C for 5 min. Next, 30 µL of 26 μM CaCl2 was added to the incubated plasma-reagent mixture. Absorbance was measured every 10 s for 8 min at 405 nm with shaking. For prothrombin time (PT) analysis, 30 µL of plasma and PT reagent (ThermoFisher Scientific, Waltham, MA, USA) were mixed, and the OD ratio was measured immediately for 8 min with a 10 s interval without shaking. Because the PT reagent already contains excess CaCl2, the coagulation reaction begins immediately. The time point with the highest value of △OD (Time(n + 1), Time(n)) was selected as the result of the PT and aPTT tests.

### 2.5. In Vivo Bleeding Test

To study clotting activity changes caused by the *F8* mutation, an in vivo bleeding test was conducted by measuring blood loss from the distal tail vein, as previously reported [14]. Briefly, mice were anesthetized via intraperitoneal (IP) injection of avertin, and 1 cm from the distal tail was cut. Blood was collected for 20 min, and the weight of the blood was normalized to the body weight of the mouse (mg/g). To determine an accurate measure of blood volume, erythrocytes were separated by centrifugation at 3000 × *g* for 10 min at RT. The supernatant was removed using a vacuum pump. The pelleted erythrocytes were re-suspended in 2 mL of RBC lysis buffer and centrifuged at 10,000 rpm for 5 min at RT. The supernatant was gently transferred into a 96-well plate. The absorbance of hemoglobin was measured using a microplate spectrophotometer at 550 nm [17]. Clotting disorder was confirmed by comparison between normal and hemophilic phenotypes.

### 2.6. Complete Blood Count

Approximately 500 µL of fresh blood was collected from the anterior vena cava using an EDTA collecting tube. Complete blood count analysis was conducted using Procyte Dx (IDEXX, Westbrook, MA, USA).

### 2.7. Calibrated Automated Thrombogram

Thrombin generation activity was measured using a Technothrombin^®^ TGA kit (Diapharma, West Chester, OH, USA). Whole blood was collected with 3.2% of sodium citrate solution, and plasma was collected after centrifugation at 1500× *g* (platelet-poor plasma) and 100× *g* (platelet-rich plasma) for 10 min at room temperature. A mixture of 50 µL of the substrate, 10 µL of reagent C low buffer, and 40 µL of 1:2 diluted plasma were mixed in a 96-well microplate. The fluorescence signal was measured for 120 min at 1 min intervals using a Cytation 5 (BioTek, Winooski, VT, USA). Thrombin generation curves were analyzed using the software that the manufacturer provided.

### 2.8. Survival Rate Analysis

Survival rates were analyzed in 8–16 week-old male C57B6 wild type (*n* = 13) and F8 I22Imice (*n* = 21) over 12 days. Bleeding was induced by toe clipping on the first day of the test. After toe clipping, excessive blood loss was prevented by electrosurgical hemostasis using a Change-A-Tip cautery DEL1 (Bovie Medical, FL, USA). Mice were monitored daily for vital conditions. The Kaplan–Meier method was used to analyze the survival rates.

### 2.9. Histological Examination

Formalin-fixed tissues were stained with hematoxylin and eosin (H&E). For H&E staining, deparaffinized tissues were stained with 0.1% Mayer’s hematoxylin and eosin (H&E) solution.

### 2.10. Statistical Analysis

Statistical analysis was performed using unpaired Student’s t-test and Mantel-Cox test using GraphPad Prism version 8.02. (GraphPad, San Diego, CA, USA).

## 3. Results

### 3.1. Potent sgRNA Selection in the F8 Gene by In Vitro Screening

**Figure 1:**
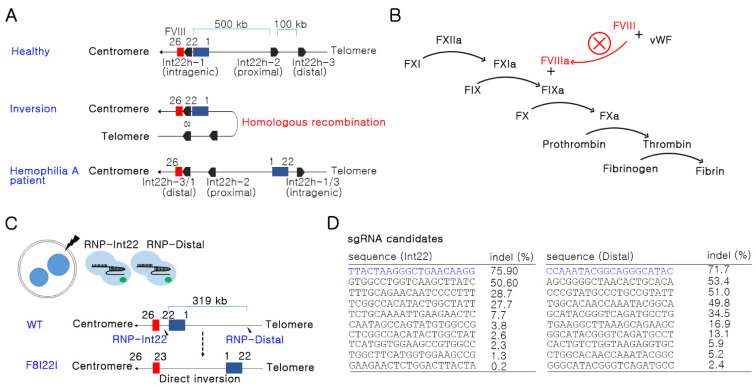
Strategy for Factor 8 intron 22 inversion (F8I22I) mouse generation. (**A**) Mechanism of F8I22I in human hemophilia A. (**B**) Brief coagulation pathway related to FVIII activation and consequent coagulation disorder. (**C**) Brief schematic for inversion-based HA model mouse generation. Two sgRNAs and Cas9 protein were transfected into mouse embryos using electroporation. The distance between the two target sites is approximately 319 kbps. Black lightning bolt: target site, blue box: *F8* exon 1–22, red box: *F8* exon 23–26. (**D**) sgRNAs with high DNA breakage potential were selected after screening using RNP transfection into NIH3T3 cell line and deep sequencing.

Human F8I22I occurs by homologous recombination between intron22 homologs: int22h-1, int22h-2, and int22h-3 at a distance of 500 kb and 575 kb (Figure 1A). Repeats of homologs induce F8I22I via non-allelic homologous recombination [13]. As a result of the F8I22I mutation, patients have a problem producing functional FVIII protein, which obstructs downstream cascades of the coagulation pathway (Figure 1B). The mouse genome does not show a complete homology to the human *F8* gene sequence. Thus, one sgRNA was designed at the 22nd intron of the *F8* gene, and the other sgRNA was designed 319 kb away from the first sgRNA in the direction of the telomere (Figure 1C). Simultaneous double-stranded DNA breakage (DSB) at the target site is essential for generating an inversion mutation. Additionally, highly efficient sgRNA is necessary for inducing inversion; thus, we selected the most efficient sgRNA candidates from each site after screening the candidates in the mouse NIH3T3 cell line by deep sequencing (Figure 1D).

### 3.2. Functional F8 Deficiency in F8I22I Mouse

**Figure 2:**
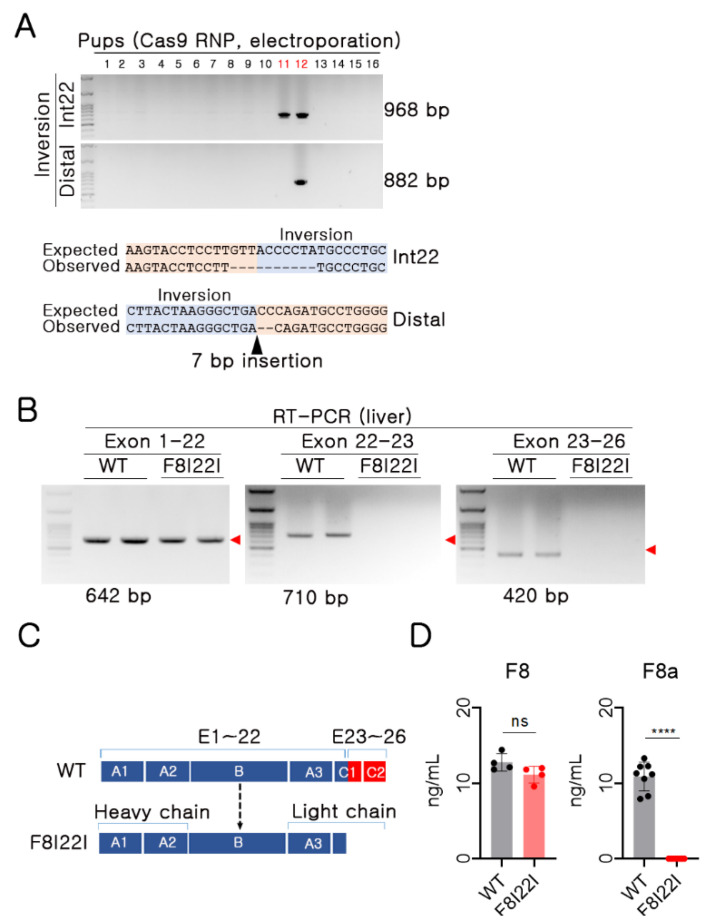
Generation of F8I22I mouse. (**A**) PCR and Sanger sequencing-based genotyping using gDNA. (**B**) RT-PCR for each mRNA fragment of exon 1–22, 22–23, and 23–26. Red symbol: target size. (**C**) Expected amino acid translation between wild type (WT) and F8I22I mouse. Blue box: preserved amino acids after inversion, Red box: missed amino acid after inversion. (**D**) Reactive protein of FVIII and FVIII activity was measured by ELISA and chromogenic analysis. Each dot indicates data from an individual mouse (FVIII ELISA, WT: *n* = 5, F8I22I: *n* = 5 and FVIII activity, WT: *n* = 8, F8I22I: *n* = 8). Data are presented as mean ± SEM. ns: not significant, ****: *p* < 0.0001.

To produce the F8I22I mutation mouse model, embryos were electroporated with Cas9 protein and two sgRNAs. As a result, 2 out of 16 pups showed F8I22I in PCR genotyping (2/16, 12.5%). One pup appeared to have breakage of DNA fragments during inversion (pup no. 11), while another pup had the intact form of F8I22I (pup no. 12). An accurate sequence of the mutants was confirmed by direct sequencing (Figure 2A). Furthermore, the mutant mice were subjected to germline transmission by breeding with WT mice, and pups from the founder were utilized for further phenotypic analysis of hemophilic symptoms.

Upon inversion being confirmed at the genomic DNA level, mRNA transcription and protein translation were also analyzed to identify the effects of the inversion. In human F8I22I hemophilia patients, amino acids of the C2 domain are synthesized by alternative small-sized mRNA of 23rd–26th exons. However, in the F8I22I mouse, no mRNA sequences derived from 23rd–26th exons were detected; even 1st–22nd exons were transcribed normally (Figure 2B). In addition, F8I22I heterozygote female mice were subjected to RT-PCR, and there was no difference in *F8* mRNA level in the liver and lung (Supplementary Figure S1).

The A3, C1, and C2 domains make up the light chains of the FVIII protein and have critical functional roles in the FVIII activity. Exons 23–26 encode amino acids in the C1 and C2 domains of the FVIII light chain; therefore, F8I22I was expected to be impaired in the light chain structural portion of the protein (Figure 2C). Similar to the results of RT-PCR, upon analyzing the FVIII protein using ELISA, which recognized the A3 domain of FVIII, F8I22I mice presented antigen levels of FVIII protein compared to WT. However, as expected, there was no detectable FVIII activity in F8I22I mice, as confirmed by the F VIIIa assay using mouse plasma (Figure 2D). These results suggest that F8I22I mice are deficient in functional FVIII production.

### 3.3. Laboratory Diagnosis for Hemophilia

**Figure 3:**
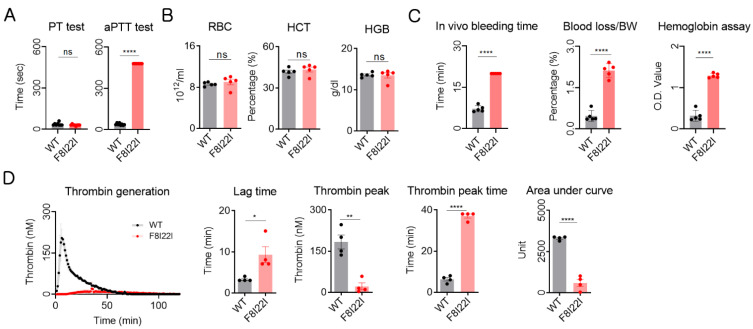
Hemophilia diagnosis using lab experiment in F8I22I. (**A**) Coagulation activity was assessed by partial thromboplastin time (PT), activated partial thromboplastin time (aPTT) (WT: *n* = 5, F8I22I: *n*=5). (**B**) Complete blood count (CBC) was conducted to analyze hemoglobin composition (WT: *n* = 5, F8I22I: *n* = 5). (**C**) In vivo bleeding analysis for confirmation of coagulation disorder (WT: *n* = 5, F8I22I: *n* = 5). (**D**) Thrombin generation potential was analyzed by the calibrated automated thrombogram using platelet-rich plasma (WT: *n* = 4, F8I22I: *n* = 4). The manufacture supplying software calculated lag time, peak height, and peak time. Each dot represents data from an individual mouse and is presented as mean ± SEM. ns: not significant, *: *p* < 0.05, **: *p* < 0.01, ****: *p* < 0.0001.

To analyze hemophilic symptoms in the transgenic hemophilic mouse, prothrombin time (PT) and activated partial thrombin time (aPTT) tests were conducted with WT and F8I22I plasma. Similar to human HA patients, there was no difference in the PT test, but F8I22I mice showed a prolonged aPTT time (Figure 3A). Next, red blood cell (RBC), hematocrit (HCT), and hemoglobin (HGB) were measured by complete blood count (CBC). There were no composition differences between WT and F8I22I mice (Figure 3B). Additionally, the in vivo bleeding time test revealed severe hemophilic disorder in F8I22I mice, as evidenced by the extended bleeding time and high blood loss as compared to the WT mouse. Since the in vivo bleeding experiment was terminated at 20 min, the blood loss per body weight in F8I22I mice was measured more than quadruple; however, the bleeding did not stop even at the end of the experiment (Figure 3C). Calibrated automated thrombogram (CAT) analysis showed that the thrombin generation activity of F8I22I mice was mostly lost (Figure 3D and Supplementary Figure S2). Taken together, these results show that a coagulation disorder similar to hemophilia was mimicked in F8I22I mice.

### 3.4. Bleeding in Lung and Liver of F8I22I Mouse

Blood coagulation disorders can induce internal hemorrhages. Human patients with HA develop internal hemorrhage most commonly in joints, and in rare complicated cases, bleeding has been observed in the brain, kidney, lung, and spleen [18,19,20,21]. To observe internal hemorrhage in F8I22I mice, H&E staining was conducted on the brain, joint, liver, kidney, lung, and spleen tissues. The F8I22I mice showed no detectable differences in the joint tissues but increased levels of internal hemorrhage in the liver and lungs of the F8I22I mice when compared to the control mice.

**Figure 4:**
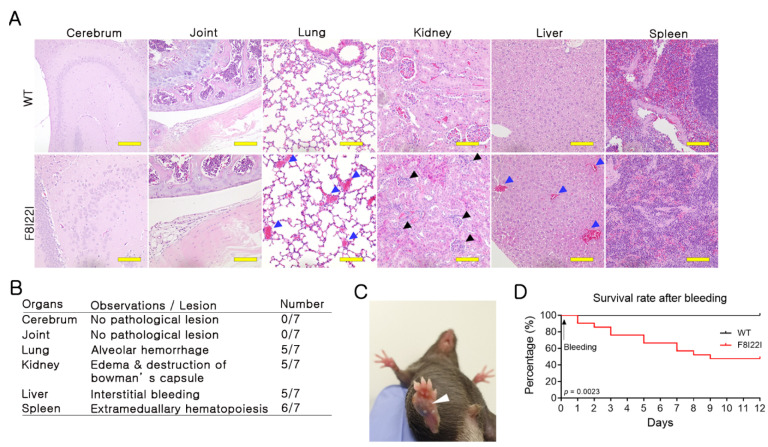
Hemophilia phenotype of F8I22I. (**A**,**B**) Histological analysis in the cerebrum, joint cavity, lung, kidney, liver, and spleen of WT and F8I22I. Black triangles indicate an edematous lesion in the kidney, and blue triangles indicate bleeding. Scale bar, 100μm. (**C**) Macroscopic observation of subcutaneous bleeding in the F8I22I. (**D**) Survival rate was analyzed in WT and F8I22I for 12 days after external bleeding (WT: *n* = 13, F8I22I: *n* = 21).

Interestingly, unexpected histological changes in the kidneys were observed. F8I22I mice exhibited characteristic edema symptoms in Bowman’s capsule, which was not detected in WT mice (Figure 4A,B). These differences could be a reflection of the intra-species difference between mice and humans. Moreover, evidence of subcutaneous hemorrhage was observed in some F8I22I mice (Figure 4C). Thus, when verifying therapy efficacy using an animal model, observations of pathological changes can be used as assessment criteria.

To confirm the severity and vitality of F8I22I-mediated hemophilia A, WT and F8I22I mice were induced for external bleeding. After bleeding, all mice were cauterized immediately to prevent exsanguination and observed every day for 12 days. All WT mice survived, but more than 50% of the F8I22I mice died during the experiments (Figure 4D). These results suggest that F8I22I-mediated HA mice exhibit severe hemophilic phenotypes.

## 4. Discussion

Hemophilia is a common, X-linked recessive genetic disease mainly caused by loss-of-function mutations in the *F8* gene. Despite the intron 22 inversion in the *F8* gene is the most frequent mutation of severe HA in humans, the development of a suitable F8I22I mouse model has not been reported till date. Thus, in this study, a mouse model harboring the inversion in the *F8* gene similar to human F8I22I was produced through consequent inversion by CRISPR/Cas9-based dual DSB formation and clinical symptoms of severe hemophilia were confirmed.

Mutations in genes induce various diseases, and animal models of genetic diseases are essential for mechanistic studies and the development of therapies. However, structural variations are difficult to fabricate through classical embryonic stem cell targeting. The first developed tool to induce inversion or translocation is a recombinase-mediated rearrangement system, such as cre-loxp22. Endonuclease can also facilitate structural variation by inducing only two DSBs on the chromosome [15,22]. Thus, we assumed that F8I22I could be generated and showed direct chromosomal inversion. Although the efficiency of gene editing was low, we successfully generated a F8I22I transgenic animal model.

The existing HA mouse model is a loss-of-function model caused by a frameshift through exon deletion [23]. However, the F8I22I mouse model is applicable to gene-editing-based therapy research because it mimics the gene structure. Recently, advanced AAV-mediated therapy has been actively studied. The effect is maintained for a longer time than prophylaxis, and the effect can last up to several years with a single injection [24]. However, HA patients with FVIII inhibitors or AAV-capsid antibodies may experience a reduction in the efficacy of the therapy. Additional drawbacks remain, such as unknown side effects due to random AAV integration [25,26]. Therefore, to permanently rescue hemophilia, gene correction treatment can be considered as the best option for patients with F8I22I-mediated HA.

A previous study has demonstrated successful functional rescue of FVIII deficiency in a mouse model by CRISPR/Cas9-mediated gene correction using HA patient-derived cells [27]. Moreover, various studies have reported the potential for gene correction in F8I22I patients [28,29]. However, HA mouse models such as the exon 16 KO and exon 17 KO models have been developed to date [10]. Although these models are well-established HA mouse models, they only exhibited loss-of-function in *F8* but did not mimic the genetic structure of patients with F8I22I. Due to these limitations, the existing animal models are difficult to utilize in F8I22I gene correction studies.

The F8I22I model developed in the current study has showed clear results in aPTT, in vivo bleeding time test, and CAT assays related to the evaluation of hemophilia symptoms. These assays are well-established experiments for therapeutic evaluation research using animal models. The pathological examination did not show the joint hemorrhage in 12~16 weeks old mice expected in humans. Although we could not observe, internal bleeding in the joint might happen at a different age or housing condition. Instead, micro-bleeding in the liver and lungs was confirmed. Pathohistological lesions in the liver, lung, spleen, and kidney were reported in some cases but not common in patients with hemophilia A [18,19,20,30]. We reasoned that histological and functional differences between humans and mice might cause this.

## 5. Conclusions

Here, we present a novel inversion-based hemophilia A mouse model. All the mice obtained by our method exhibited stable laboratory test results that showed symptoms of severe coagulation disorder. This structural variation-based disease animal model will be applicable not only to the evaluation of general hemophilia drugs but also to the development of gene-editing-based therapy research.

## Data Availability

All relevant data are included within the manuscript and supplementary file. The raw data are available on request from the corresponding author.

## Supplementary Materials

The following are available online at https://www.mdpi.com/article/10.3390/biology10080704/s1. Figure S1: Thrombin generation potential was analyzed by the calibrated automated thrombogram using platelet-poor plasma (WT: *n* = 4, F8I22I: *n* = 4). Lag time, peak height, and peak time were calculated by the manufacture supplying software. Each dot represents data from an individual mouse and is presented as mean ± SEM. **: *p* < 0.01, ***: *p* < 0.001, ****: *p* < 0.0001, Figure S2: RT-PCR for each mRNA fragment of exon 1–22, 22–23, and 23–26 using mRNA from F8I22I heterozygote female mouse. Red symbol: target size,
