# Supplementary material for: Novel Severe Hemophilia A Mouse Model with Factor VIII Intron 22 Inversion
Source: Biology (Basel). 2021 Jul 23;10(8):704. doi: 10.3390/biology10080704 (PMC8389204; doi:10.3390/biology10080704)
Supplement: Supplementary file 1 [file biology-10-00704-s001.zip › biology-1280891-supplementary.pdf]

Supplementary information for

Novel severe hemophilia A mouse model with Factor VIII intron 22 inversion

Authors

Jeong Pil Han[1], Dong Woo Song[2], Jeong Hyeon Lee[1], Geon Seong Lee[1], Su Cheong Yeom[1,3,]*

Corresponding authors

Su Cheong Yeom, DVM, Ph.D., Associate professor, Graduate School of International Agricultural Technology, Seoul National University, 1447 Pyeongchang-Ro, Daewha, Pyeongchang, Gangwon 25354, Korea

Tel: 82-33-339-5750, Fax: 82-33-339-5762, E-mail: scyeom@snu.ac.kr

This file includes

1. RT-PCR image for F8I22I heterozygote female mouse
2. Thrombin generation potential analysis using platelet-poor plasma

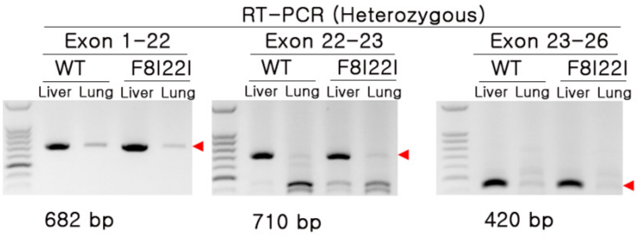

Figure S1. RT-PCR for each mRNA fragment of exon 1-22, 22-23, and 23-26 using mRNA from F8I22I heterozygote female mouse. Red symbol: target size.

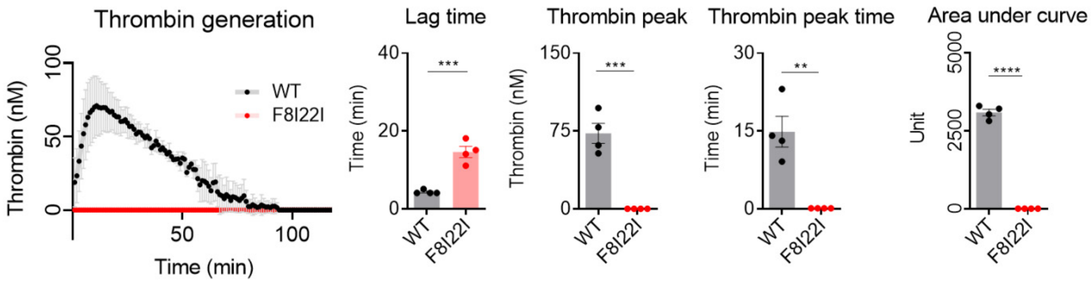

Figure S2. Thrombin generation potential was analyzed by the calibrated automated thrombogram using platelet-poor plasma (WT: n=4, F8I22I : n=4). Lag time, peak height, and peak time were calculated by the manufacture supplying software. Each dot represents data from an individual mouse and is presented as mean ± SEM. ** : $p < 0.01$, ***: $p < 0.001$, ****: $p < 0.0001$
